# Development and in vivo validation of tissue-engineered, small-diameter vascular grafts from decellularized aortae of fetal pigs and canine vascular endothelial cells

**DOI:** 10.1186/s13019-017-0661-x

**Published:** 2017-11-25

**Authors:** Xu Ma, Zhijuan He, Ling Li, Guofeng Liu, Qingchun Li, Daping Yang, Yingbo Zhang, Ning Li

**Affiliations:** 1 Department of Plastic Surgery, The Second Affiliated Hospital of Harbin Medical University, 246 Xuefu Road, Nangang District, Harbin, Heilongjiang 150086 China; 2 Department of Obstetrics and Gynecology, The First Affiliated Hospital of Harbin Medical University, 23 Youzheng Street, Nangang District, Harbin, Heilongjiang 150086 China; 3 Department of Cardiology, The Second Affiliated Hospital of Harbin Medical University, 246 Xuefu Road, Nangang District, Harbin, Heilongjiang 150086 China

**Keywords:** Vascular endothelial cells, Decellularized aortae of fetal pigs, Scaffold, Tissue-engineered small-diameter vascular grafts

## Abstract

**Background:**

Tissue engineering has emerged as a promising alternative for small-diameter vascular grafts. The aim of this study was to determine the feasibility of using decellularized aortae of fetal pigs (DAFPs) to construct tissue-engineered, small-diameter vascular grafts and to test the performance and application of DAFPs as vascular tissue-engineered scaffolds in the canine arterial system.

**Methods:**

DAFPs were prepared by continuous enzymatic digestion. Canine vascular endothelial cells (ECs) were seeded onto DAFPs in vitro and then the vascular grafts were cultured in a custom-designed vascular bioreactor system for 7 days of dynamic culture following 3 days of static culture. The grafts were then transplanted into the common carotid artery of the same seven dogs from which ECs had been derived (two grafts were prepared for each dog with one as a backup; therefore, a total of 14 tissue-engineered blood vessels were prepared). At 1, 3, and 6 months post-transplantation, ultrasonography and contrast-enhanced computed tomography (CT) were used to check the patency of the grafts. Additionally, vascular grafts were sampled for histological and electron microscopic examination.

**Results:**

Tissue-engineered, small-diameter vascular grafts can be successfully constructed using DAFPs and canine vascular ECs. Ultrasonographic and CT test results confirmed that implanted vascular grafts displayed good patency with no obvious thrombi. Six months after implantation, the grafts had been remodeled and exhibited a similar structure to normal arteries. Immunohistochemical staining showed that cells had evenly infiltrated the tunica media and were identified as muscular fibroblasts. Scanning electron microscopy showed that the graft possessed a complete cell layer, and the internal cells of the graft were confirmed to be ECs by transmission electron microscopy.

**Conclusions:**

Tissue-engineered, small-diameter vascular grafts constructed using DAFPs and canine vascular ECs can be successfully transplanted to replace the canine common carotid artery. This investigation potentially paves the way for solving a problem of considerable clinical need, i.e., the requirement for small-diameter vascular grafts.

## Background

Coronary artery and peripheral arterial diseases have high rates of mortality and morbidity and so represent a massive economic and clinical burden to healthcare worldwide [1]. The most promising approach to solving this vascular problem and thus reducing the morbidity associated with these diseases is the use of small-diameter (<6 mm) vascular grafts [2]. Although autologous vessels (e.g., saphenous veins) represent the gold standard grafts for small-diameter vessels, many patients do not have veins suitable for grafting [3]. Thus, there is a considerable clinical need for small-diameter vascular grafts.

Tissue engineering has emerged as a promising alternative for producing small-diameter vascular grafts [4]. Tissue engineering strategies consist of three main components: scaffolds that house the cells and support cellular growth and activity; seed cells, which preserve the specific function of the tissue; and a nurturing environment [5]. Scaffolds provide temporary or permanent support to damaged tissues, and scaffold materials can be generally divided into two categories: native biological materials and synthetic polymeric materials [6]. Compared with synthetic polymer-based scaffolds, natural polymers present a biologically active environment to cells and promote excellent cell adhesion and growth [7]. However, numerous studies have also reported on the poor mechanical properties of natural polymers [7–9]. Recently, decellularized tissue-engineered vascular grafts have been widely used as natural scaffolds to produce arterial conduits that provide ideal biomechanical properties and cell compatibility [10, 11]. For instance, Böer et al. showed that intensified decellularization of equine carotid arteries generated highly suitable matrix scaffolds for vascular tissue engineering [12]. However, the in vivo application of tissue-engineered vascular grafts has not been widely investigated.

Recently, Liu et al. studied the mechanical properties of decellularized aortae of fetal pigs (DAFPs) and conducted an assessment of cell adhesion and compatibility by seeding with porcine aortic endothelial cells (ECs) and performing subdermal implantation in adult male Sprague Dawley rats [13]. Their results showed that DAFPs exhibited minimal calcification and exhibited almost no immunological reaction during the entire follow-up period [13]. In this study, small-diameter vascular grafts were constructed with DAFPs and by seeding with canine vascular ECs. These vascular grafts were then implanted into the same dogs from which ECs had been derived. The aim of this study was to determine the feasibility of using DAFPs to construct tissue-engineered small-diameter vascular grafts and to test the performance and application of DAFPs as tissue-engineered vascular scaffolds in the canine arterial system.

## Methods

### Experimental animals

Fetal pigs of 100-day gestational age were delivered by cesarean section. Adult mongrel dogs were used as both vascular EC donors and graft recipients. All animals received humane care in compliance with the Guide for Care and Use of Laboratory Animals published by the National Institutes of Health (NIH publication No. 85-23, revised 1996).

### Preparation of fetal pig aorta

Pregnant sows were intravenously anesthetized with 1% pentobarbital sodium (10 mg/kg) and delivered by cesarean section. After removal of fetuses, sows were euthanized by excessive anesthesia. Fetal pigs with crown-rump lengths of 25–30 cm were selected and 5-cm sections of aorta were excised under sterile conditions. These specimens werecryopreserved immediately transported to the laboratory with a warm ischemia time of <30 min [14].

### Preparation of DAFPs

Fetal pig aortae with an outer diameter of 4 mm and a length of 6 cm were selected. After removal of surrounding tissues under sterile condition, the blood vessels were washed with sterile saline several times to remove residual blood. DAFPs were prepared by continuous enzymatic digestion using trypsin, DNase, and RNase as previously described [15] with few modifications. Aortae were first digested with a solution containing 0.1% trypsin/0.02% EDTA in phosphate-buffered saline (PBS) (without Ca^2+^ and Mg^2+^) for 36 h (all reagents from Sigma, St. Louis, Mo., USA), and the solution was changed every 12 h. The aortae were then decellularized with 20 μg/mL RNase and 200 μg/mL DNase (Boehringer, Mannheim, Germany) for 4 h at 37 °C under a humidified atmosphere of 5% CO_2_ and 95% air with constant gentle shaking. DAFPs were then washed with sterile PBS several times to remove the digestion residue. Following DAFP preparation, approximately 3 mm of the tissue at the end of each decellularized specimen was taken and cut into two semi-rings. Half of the tissue was examined following conventional hematoxylin–eosin (HE) staining, and the other half was used for DNA quantification [16]. Finally, DAFPs were subjected to vacuum freeze-drying and ethylene oxide sterilization.

### Culture and identification of canine vascular ECs

Seven domestic dogs aged 6 months and weighing 25 kg were used. Intravenous administration of 1% pentobarbital sodium (10 mg/kg) was used for anesthesia. Approximately 10 cm of the left external jugular vein was excised under aseptic condition as previously described [17]. Primary vascular ECs were obtained by enzymatic digestion as previously described [18]. In brief, the outer membrane layer of the vessel was removed and the vessel was cut longitudinally to form a sheet. The intima layer was flattened on the culture dish. A solution of 0.2% collagenase A (Boehringer Mannheim, Germany) in PBS with Ca^2+^ and Mg^2+^ was injected beneath the inner membrane surface and subsequent digestion performed in an incubator for 15 min. The digested EC suspension was collected and centrifuged at 1000 rpm for 5 min. The EC pellet was resuspended and cultured in medium 199 (GIBCO) supplemented with 10% fetal bovine serum, 100 units/mL penicillin (Sigma), 100 mg/mL streptomycin (Sigma), 0.25 mg/mL amphotericin B (Sigma), 5 ng/mL endothelial growth factor (Boehringer Mannheim) and 1% L-glutamine (Sigma). Cells were passaged using trypsin digestion and the fourth generation was used as seed cells (the number of vascular seed cells in the fourth generation was sufficient for seeding purposes, and ECs still possessed abundant proliferative capacity at this passage). Cells were confirmed to be vascular ECs by morphology as well as by immunohistochemistry and immunofluorescence to detect the endothelial marker von Willebrand factor (vWF) as previously described [19].

### Construction of tissue-engineered, small-diameter vascular grafts

**Fig. 1:**
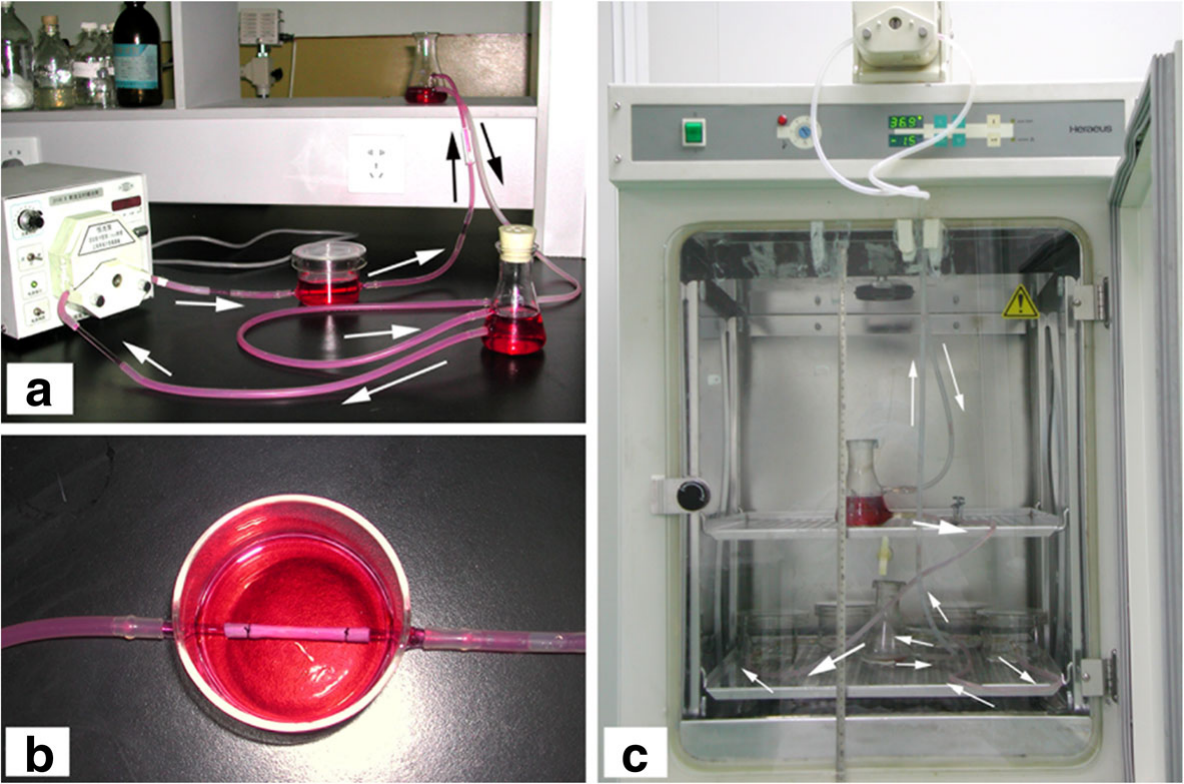
A custom-designed and -produced vascular bioreactor system. **a**, the design of the vascular bioreactor system. **b**, the processing chamber used for specimen fixation. **c**, operation of the dynamic vascular bioreactor system. The dynamic vascular bioreactor system was placed into a tissue culture incubator. The height difference between culture solutions was regulated to control the static pressure within the vascular grafts. A controlled peristaltic pump regulated the dynamic pressure within the vascular grafts and the flow rate variation of the culture solutions. The arrows show the operation of the dynamic vascular bioreactor system

Vacuum freeze-dried DAFP material was soaked in sterile PBS for 24 h. Vascular ECs were seeded onto DAFPs by rotational precipitation. Briefly, ECs were trypsinized to form a cell suspension and the EC concentration was adjusted to 3 × 10^6^ cells/mL. Both ends of DAFP tubular stents were clamped and the cell suspension was injected into the arterial lumen using a syringe. The arteries were then rotated 120° after standing for 10 min and seeding was completed after a total rotation of 360°. The grafts were transferred to a vascular bioreactor system for 7 days of dynamic culture following 3 days of static culture. A vascular bioreactor system was designed and produced by our research group as depicted in Fig. 1. This bioreactor system was driven by a peristaltic pump [20] and specimens were placed in the processing chamber (Fig. 1b). The liquid flow in the system was gradually increased by adjusting the speed of the peristaltic pump so that the perfusion rate increased from 20 to 60 mL/min (the perfusion rate was 20 mL/min on the first day of dynamic culture and increased by 10 mL/min daily to 60 mL/min on the fifth day of dynamic culture, at which point the perfusion rate was maintained at 60 mL/min through to the seventh day). The static pressure due to height differences of the culture solutions was 10 mmHg, and the dynamic pressure generated by the peristaltic pump was 60 mmHg. After culture completion, the fixed ends (lacking ECs) were removed and then the grafts were used for surgical implantation into the same dogs from which ECs had been derived.

### Surgical implantation of vascular grafts

The same seven dogs from which left external jugular veins were harvested to obtain ECs received general anesthesia. Routine preoperative preparation was followed and the right common carotid artery was exposed. A location of the carotid artery with an external diameter of approximately 4 mm at diastole was selected as the site for implantation of the tissue-engineered vascular grafts. Vascular clamps were placed on both sides of the selected position, and the artery (4 mm in diameter) was transected. The tissue-engineered vascular grafts were used to bridge the common carotid artery using microsurgical techniques. The incisions were closed, and routine intramuscular injection of heparin as well as intravenous injection of antibiotics were applied postoperatively. Doppler ultrasonography was conducted at 1, 3, and 6 months and contrast-enhanced CT at 6 months, following implantation. Vascular grafts were sampled for histological and electron microscopic examination after the dogs were sacrificed at 6 months post-implantation.

### Doppler ultrasonography and enhanced CT examination

All experimental animals received general anesthesia prior to each procedure and neck skin preparation prior to ultrasonic examination. Bilateral common carotid artery ultrasound was performed using a Doppler ultrasound device. Animals were injected with contrast medium prior to three-dimensional CT angiography.

### HE staining

DAFPs and sampled vascular grafts were fixed in 10% neutral-buffered formalin solution, embedded in paraffin, transversely sectioned (5 μm) and stained using HE for histological assessment of general morphology.

### Electron microscopy

Specimen surface ultrastructure was examined by scanning electron microscopy (SEM). Briefly, specimens were sequentially fixed in 1% (*v*/v) buffered glutaraldehyde and 0.1% (v/v) buffered formaldehyde for 1 and 24 h, respectively, dehydrated with a graded ethanol series, and dried. Dried samples were mounted on aluminum chucks and sputter-coated with gold (Cressington 108; Cressington Scientific Instruments, PA, USA). An S-3400 N scanning electron microscope (Hitachi, Tokyo, Japan) was used for sample examination.

For transmission electron microscopy (TEM), specimens were fixed in 2.5% glutaraldehyde solution, rinsed with 0.1 M phosphate buffer, and then fixed in 1% osmic acid for 2 h. Subsequent rinsing in double-distilled water was followed by dehydration in a graded series of water–acetone solution. Specimens were then impregnated with Epon 812 resin, embedded, and polymerized. Semi-thin sections were counterstained with azure II and basic fuchsin and examined with light microscopy to determine orientation. Ultra-thin sections were stained with uranyl acetate and lead citrate and then examined with a Zeiss EM10 electron microscope (Zeiss, Oberkochen, Germany).

## Results

### Identification of canine vascular ECs and morphology of DAFPs

**Fig. 2:**
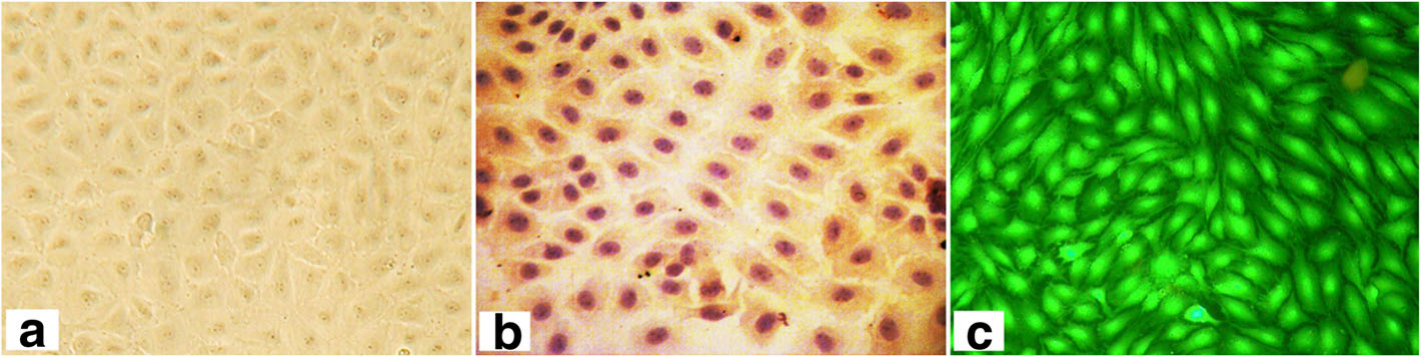
Culture and identification of canine vascular endothelial cells (ECs). **a** optical microscopy image of ECs; **b** immunohistochemical staining for von Willebrand factor (vWF); **c** immunofluorescence of vWF. Most cells were vWF-positive

Immunohistochemistry and immunofluorescence showed positive vWF staining in most cells (Fig. 2).

**Fig. 3:**
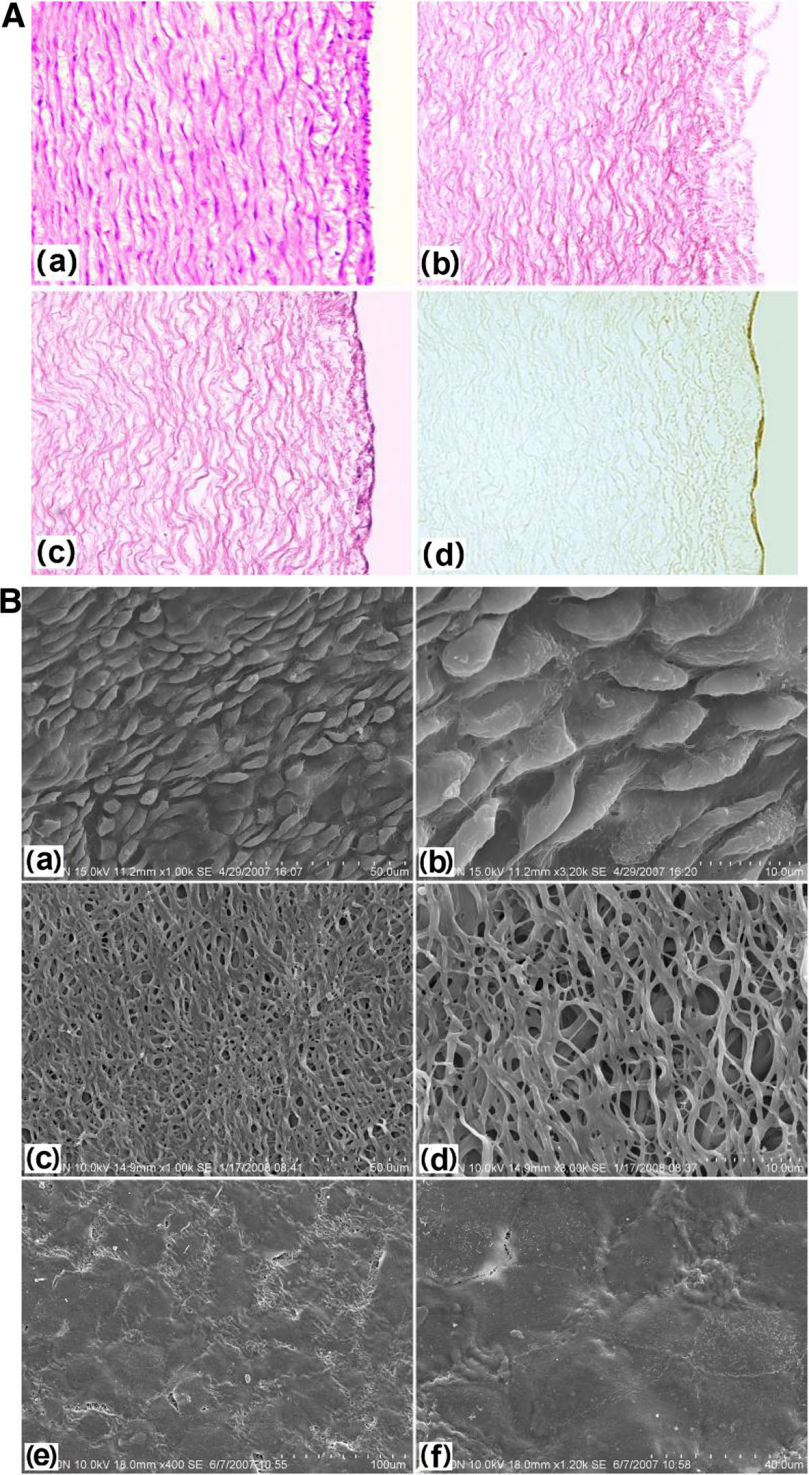
Histological and immunohistochemical staining and scanning electron microscopy of specimens. **A**: **a**, histological staining of fetal pig aorta; **b**: histological staining of decellularized aortae of fetal pigs (DAFPs); **c**: histological staining of tissue-engineered vascular grafts with an EC layer; **d**: immunohistochemical staining of ECs of vascular grafts. **B**: **a**: fetal pig aortic EC layer at low magnification; **b**: fetal pig aortic EC layer at high magnification; **c**: the inner elastic membrane of DAFPs at low magnification; **d**: the inner elastic membrane of DAFPs at high magnification; **e**: the EC layer of tissue-engineered vascular grafts at low magnification; **f**: the EC layer of tissue-engineered vascular grafts at high magnification

Histological staining of fetal porcine aortae revealed a typical three-layer structure of the arteries (Fig. 3A-a), and SEM demonstrated a complete layer of ECs with a cobblestone phenotype (Fig. 3B-a, b). The DNA content of DAFPs was <0.1%. Histological staining of DAFPs showed that the extracellular matrix remained intact; however, no nuclei or intact cells were present (Fig. 3A-b). In addition, SEM examination showed that after decellularization, endogenous ECs were completely removed and the structure of the inner elastic membrane was clearly visible (Fig. 3B-c, d).

### Characterization of tissue-engineered, small-diameter vascular grafts

**Fig. 4:**
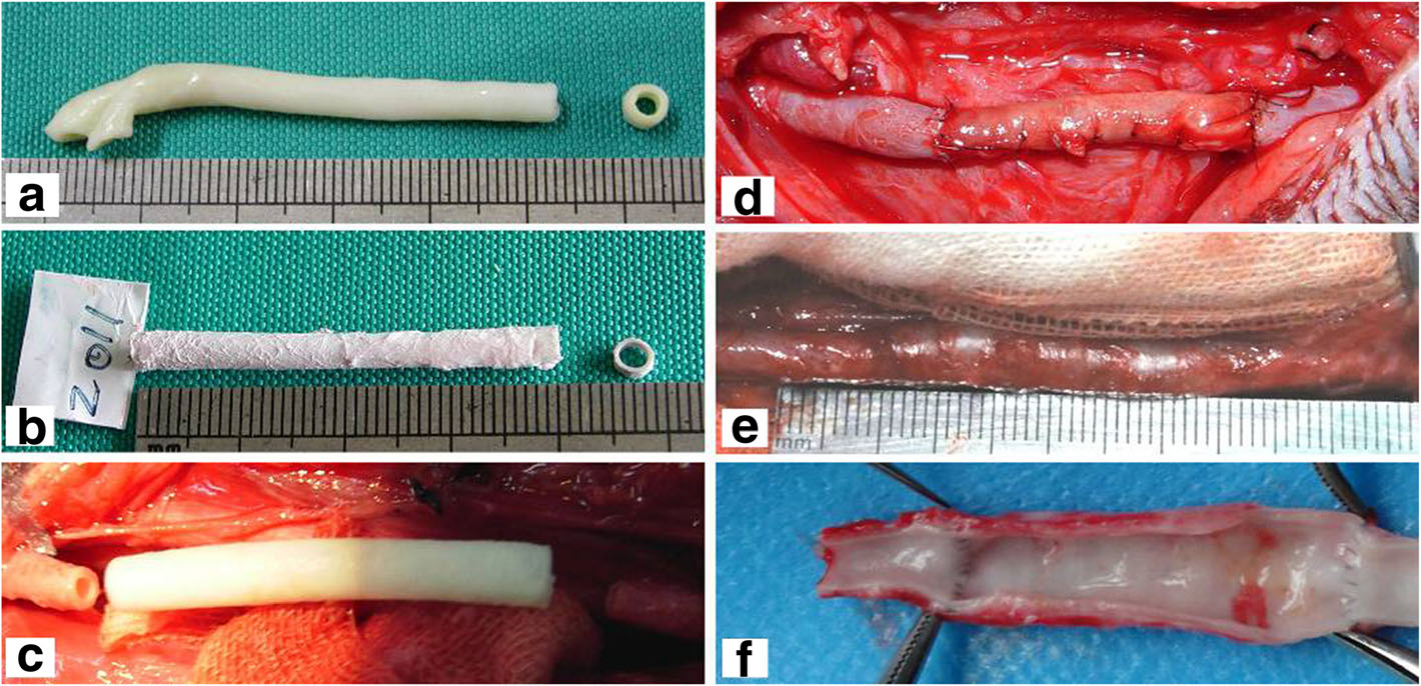
General morphology of specimens. **a** general morphology of fetal pig aorta; **b** general morphology of vacuum freeze-dried DAFPs; **c** tissue-engineered vascular grafts after dynamic seeding of ECs; **d** the implantation of tissue-engineered vascular grafts in dogs; **e** general morphology of sampled vascular grafts at 6 months post-implantation; **f** the inner morphology of sampled vascular grafts at 6 months post-implantation

After 1 week of dynamic culture in the custom-made dynamic vascular bioreactor system, vascular grafts were successfully constructed using DAFPs and canine vascular ECs (Fig. 4). The vascular grafts possessed a complete, intact EC layer. Histological findings showed that a continuous cell layer was present on the inner surface of the grafts (Fig. 3A-c), and SEM revealed that ECs were tightly connected, forming a continuous cell layer that completely covered the inner elastic membrane (Fig. 3B-e, f).

### Implantation and evaluation

**Fig. 5:**
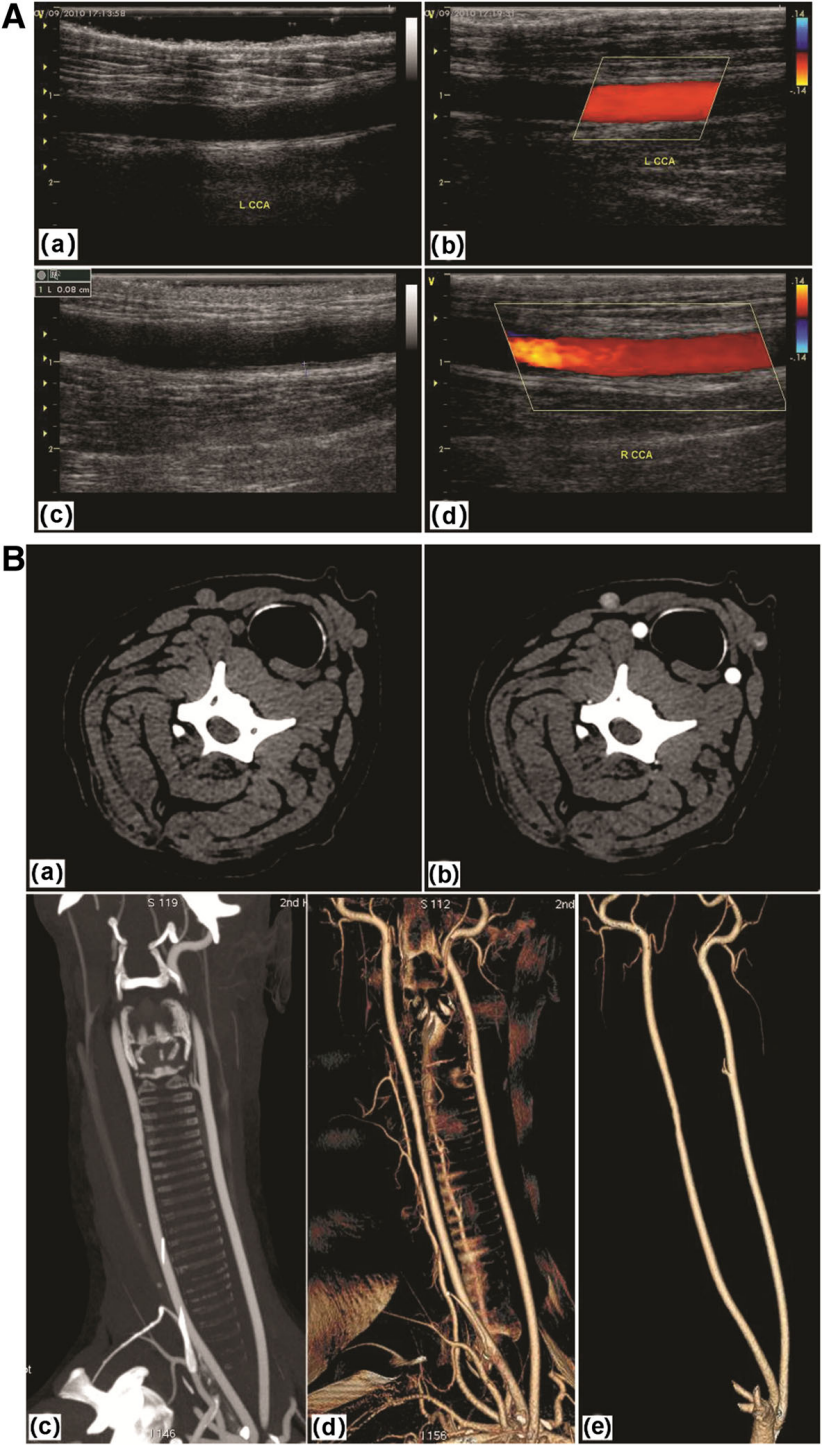
Color Doppler ultrasonography and three-dimensional CT detection after the implantation of tissue-engineered vascular grafts into canine common carotid arteries. **A**: **a** and **b**, Color Doppler ultrasonography at 3 months post-implantation; **c** and **d**, 6 months post-implantation. **B**, three-dimensional CT detection at 6 months post-implantation. **a** and **b** are horizontal images; **c** is a coronal plane image; **d** and **e** are three-dimensional images

Seven dogs were numbered and all were subjected to extensive postoperative evaluation (Table 1). Vascular diameter of the grafts matched that of the common carotid artery into which they had been implanted (Fig. 4). Following implantation, vascular grafts exhibited good patency and no obvious thrombi were found attached to the vascular wall, as assessed by Doppler ultrasound and enhanced CT examination (Fig. 5). Six months after implantation, the grafts showed no obvious stenosis and expansion. Additionally, dynamic Doppler sonography revealed good graft compliance, and vascular grafts underwent spontaneous rhythmic vasodilation and contraction in synchrony with heartbeat.

**Table 1:** Extensive evaluation of seven dogs at 6 months post-implantation of tissue-engineered vascular grafts

Number	Gross observations	Histological staining	Color Doppler ultrasonography and three-dimensional CT	Scanning electron microscopy	Transmission electron microscopy
1	No obvious expansion; smooth surface without thrombi when sampled	Similar to natural arterial structure; a continuous cell inner layer; dense tunica media	Good patency; no obvious thrombi; no obvious stenosis and expansion	Continuous endothelial cell layer; tight cell junctions	Confirmation of the continuous endothelial cell layer; identification of Weibel–Palade bodies
2	√	√	√	√	√
3	√	√	√	√	√
4	√	√	√	√	√
5	√	√	√	√	√
6	√	√	√	√	√
7	√	√	√	√	√

All dogs were tested in the same manner and “√” indicates the same positive findings

**Fig. 6:**
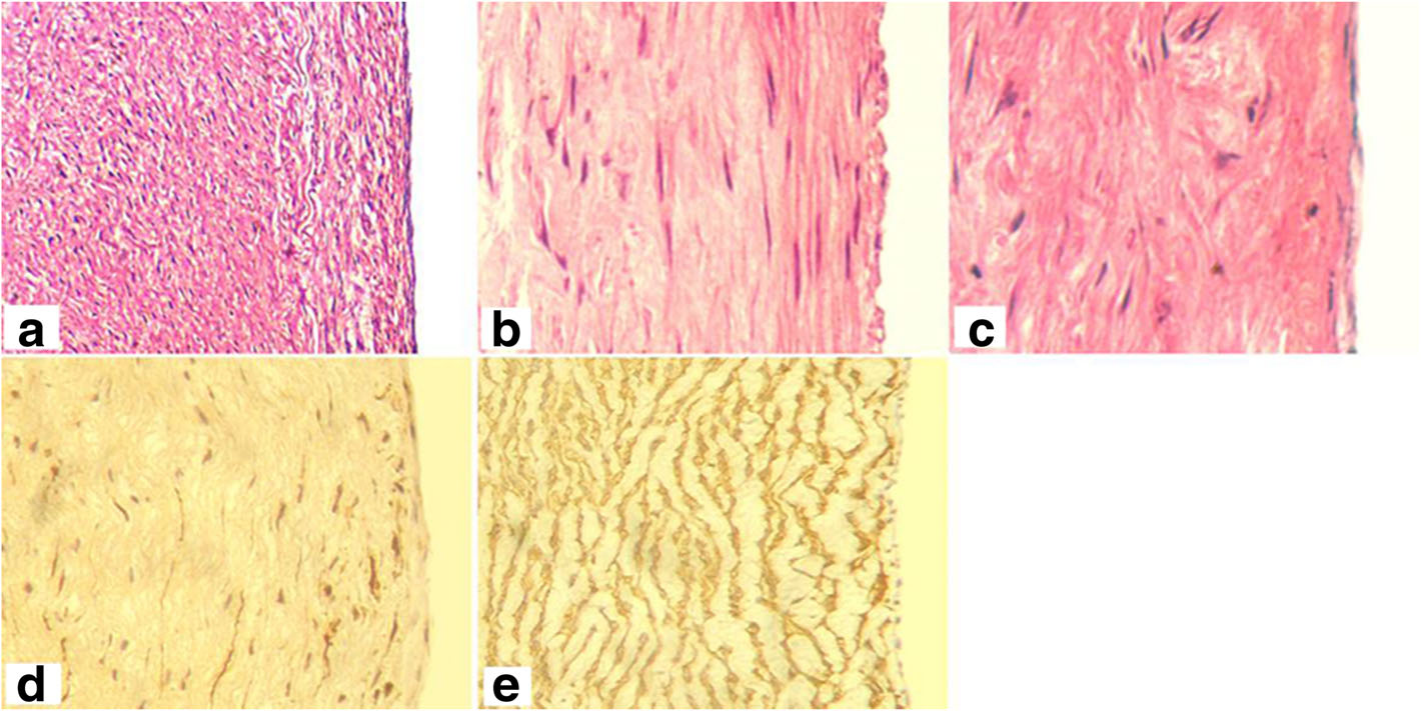
Histological staining of tissue-engineered vascular grafts after implantation. **a**, **b**, and **c**, Hematoxylin–eosin staining of tissue-engineered vascular grafts at 6 months post-implantation; **d** smooth muscle actin (SMA) immunohistochemical staining of vascular grafts; **e** SMA immunohistochemical staining of fetal pig aorta as a positive control

**Fig. 7:**
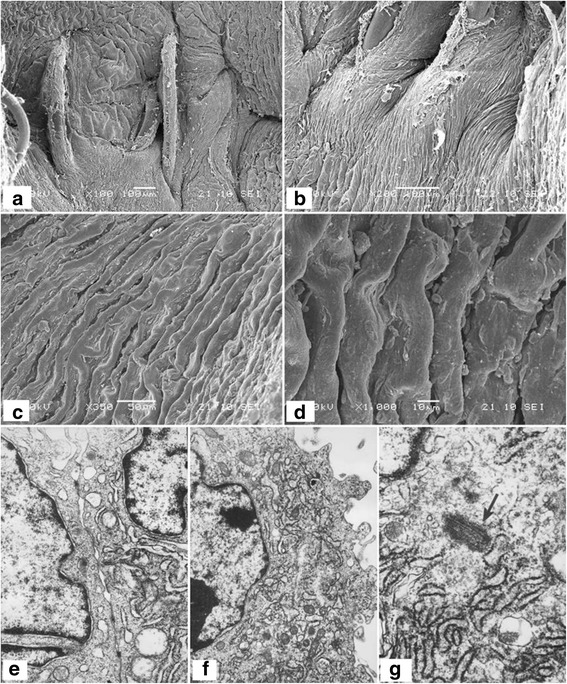
Electron microscopy of tissue-engineered vascular grafts at 6 months post-implantation. **a**–**d**, the inner surface of vascular grafts by scanning electron microscopy; **e**, close connections between ECs were identified by transmission electron microscopy (TEM). **f**, the cell cytoplasm was rich in active organelles identified by TEM. **g** specific structures of ECs as seen at high magnification by TEM

Sampling results at 6 months post-transplantation showed that the tissue-engineered vascular grafts had smooth surface and no thrombi. As depicted in Fig. 6, histological staining showed that the grafts had been remodeled within the animals, and a continuous inner cell layer was identified. The tunica media was structurally dense and similar to natural arterial structure, and cells had evenly infiltrated and were identified as muscular fibroblasts by immunohistochemistry (smooth muscle actin expression was used as a classical marker for myofibroblasts). In addition, TEM examination of the inner cells showed that their surfaces were rich in finger, spherical, and villous protrusions and that they contained abundant cytoplasmic organelles, indicating that they were indeed ECs (Fig. 7). Moreover, Weibel–Palade bodies, which are membrane-enclosed rod-shaped organelles specifically found in ECs, were also identified.

## Discussion

Extracellular matrix (ECM) scaffolds for vascular tissue engineering have mainly been derived from decellularized vascular matrix and decellularized small intestinal submucosa [21, 22]. Suitably decellularized blood vessels have the ideal shape and they possess biomechanical properties particularly suited for use as vascular grafts [23]. Li et al. have demonstrated that due to its low immunogenicity and optimal properties, decellularized fetal porcine vascular tissue could be used for tissue-engineered, small-diameter vascular grafts and as a potential alternative to xenogeneic transplantation [24]. Taking into account these studies, we hypothesized that DAFPs could be used as a vascular tissue-engineered scaffold in the canine arterial system.

ECs seeded at the blood interface can prevent vascular grafts from being directly exposed to the bloodstream, consequently preventing thrombosis on the grafts, thus increasing their patency [25]. Cell-seeding methods include static seeding with biological glues and direct seeding [26]. Dynamic seeding can increase both cell-seeding efficiency and penetration of the scaffold [26]. In this study, a custom-designed and -produced vascular bioreactor system was used for dynamic seeding. This vascular bioreactor system can simulate the internal vascular mechanical environment, promote adhesion and proliferation of ECs, and prevent seeded ECs from washing away after transplantation into the arterial system. The results of the present study showed that dynamic seeding caused ECs to tightly attach to DAFPs.

ECs and smooth muscle cells are the main cellular components of the vasculature [27]. Vascular smooth muscle cells in the tunica media have important functional roles, for instance, regulation of vascular diameter, increase of vascular compliance, and secretion of ECM to affect the function of ECs [28]. Generating a functional smooth muscle layer is therefore important for successful vascular tissue engineering. A matching level of compliance between tissue-engineered, small-diameter vascular grafts and recipient vessels determines the long-term patency of blood vessels [29]. If a compliance mismatch occurs, fluid mechanical effects, such as the hydrodynamic shear stress of blood flow, result in graft wall thickening or expansion and formation of graft occlusions or aneurysms, ultimately leading to the failure of the implant [30].

However, the native architecture and low porosity of decellularized vessels have impeded efforts to seed smooth muscle layer into tissue-engineered blood vessels [31]. We also failed to seed smooth muscle cells into DAFPs due to the relatively compact structure of decellularized vessel matrix. Our laboratory is exploring effective methods for seeding smooth muscle cells into the acellular vascular matrix in vitro and trying to improve seeding efficiency using multi-needle, micro-needle injection. Thus, in this study, tissue-engineered DAFP scaffolds were only seeded with ECs. We found that the vascular grafts we constructed in this study possessed a complete EC layer, indicating that our dynamic vascular bioreactor system meets the requirements of dynamic seeding of ECs.

We tried a variety of methods to label ECs in vitro to track seeded cells; however, our results were unsatisfactory and so cell-tracking experiments were not performed. We also implanted vascular grafts that had not been seeded with ECs in vitro, and this experiment is still ongoing. This ongoing investigation indicates that the patency of vascular grafts without seeded ECs is much lower (roughly no more than 60%) than those containing seeded ECs as described here (data not shown). The presence of an EC layer was confirmed in the vicinity of the anastomotic stoma of unobstructed vascular grafts, but the EC layer was notably absent in the middle of the grafts, indicating that ECs were migrating along the grafts from both sides of the normal blood vessel. Thus, it seems likely that in vitro seeded ECs in this study were involved in the reconstruction of the EC layer of vascular grafts. Taken together, it can be deduced that there were three sources of ECs in the inner layers of the vascular grafts: in vitro seeded ECs; normal vascular ECs migrating from both ends of the graft; and endothelial progenitor cells deposited by the blood.

The diameter of the tissue-engineered blood vessels constructed using DAFPs matched that of the canine common carotid artery. The implanted tissue vascular grafts exhibited good patency, and no obvious thrombi were found attached to the vascular walls by Doppler ultrasound and enhanced CT examination.. Taken together, our results indicated that these tissue-engineered blood vessels containing intact EC layers but no smooth muscle cell layers functioned well and were remodeled in vivo.

In this study, we examined the feasibility of using DAFPs as a heterologous biomaterial. The ultimate goal of this work is to apply this technique to clinical practice. Following further improvements and refinement of the preparation method, vascular ECs from human patients could be seeded onto this heterologous scaffold material and cultured in vitro prior to implantation back into the cell donor. However, it is clear that much research must be conducted before this technology can be applied to humans. For example, the source of vascular ECs must be the recipient of vascular grafts to avoid transplant rejection [32]. However, obtaining adult ECs from recipients is not necessarily an ideal method because of the limited number of ECs that can be acquired and the adverse effects on the recipient of additional surgical trauma. Thus, the ideal source of seed cells for tissue-engineered vascular grafts is in vitro culture and induced differentiation of bone marrow stromal stem cells or circulating stem cells from the recipient.

There are many advantages of using pigs as heterologous organ donors in xenotransplantation research [33, 34]. Fetal pig was selected as the source of a tissue-engineered vascular scaffold in this study was selected. A more standardized DAFP material and convenient method of acquisition of autologous vascular seed cells in the future may make it easier to prepare tissue-engineered vascular grafts that have similar histological structures and physiological functions to human blood vessels. This investigation potentially paves the way to addressing the considerable clinical need for small-diameter vascular grafts. However, several limitations of our study should also be considered. First, the observation time was relatively short and a longer postoperative follow-up period would be desirable. Second, a control arm lacking EC seeding has not been reported here but is currently underway. Finally, the vascular bioreactor system in this study needs to be improved to better simulate the in vivo vascular mechanics of the arterial system and to improve culture efficiency of the grafts.

## Conclusions

Tissue-engineered, small-diameter vascular grafts with an intact EC layer can be successfully constructed using DAFPs. These tissue-engineered blood vessels can be transplanted to replace the canine common carotid artery. Additionally, the grafts are structurally and functionally similar to normal artery after remodeling in vivo in animals. Therefore, DAFPs hold the potential for use in constructing tissue-engineered, small-diameter vascular grafts.

## Abbreviations

DAFP: Decellularized aorta of fetal pigs
ECM: Extracellular matrix
ECs: Endothelial cells
HE: Hematoxylin and eosin
SEM: Scanning electron microscopy
SMA: Smooth muscle actin
TEM: Transmission electron microscopy
vWF: von Willebrand factor
W-P: Weibel-Palade
